# Culturable Human Microorganisms and the Impact of Transportation Conditions on Cultivability

**DOI:** 10.3390/microorganisms13030549

**Published:** 2025-02-28

**Authors:** Xibei Fan, Ning Lv, Zhexue Quan

**Affiliations:** Microbiome Center, Shanghai Engineering Research Center of Industrial Microorganisms, School of Life Sciences, Fudan University, Shanghai 200438, China; 20110700152@fudan.edu.cn (X.F.); 22210700041@fudan.edu.cn (N.L.)

**Keywords:** human microbiology, culturomics, high-throughput sequencing, transportation conditions, protective solution

## Abstract

The composition of the human microbiome is a critical health indicator, and culture-independent methodologies have substantially advanced our understanding of human-associated microorganisms. However, precise identification and characterization of microbial strains require culture-based techniques. Recently, the resurgence of culturomics, combined with high-throughput sequencing technology, has reduced the high labor demand of pure culture methods, facilitating a more efficient and comprehensive acquisition of culturable microbial strains. This study employed an integrated approach combining culturomic and high-throughput sequencing to identify culturable microorganisms on the human scalp and in human saliva and feces. Several *Staphylococcus* strains were identified from the scalp, whereas anaerobic microorganisms were dominant in the saliva and fecal samples. Additionally, the study highlighted the beneficial effects of transportation conditions (liquid nitrogen treatment, dry ice transport, and dimethyl sulfoxide [DMSO] buffer) in preserving culturable microorganisms. A robust methodology was developed for the large-scale acquisition of culturable microorganisms with optimized transport conditions that enhance the potential for isolating a greater diversity of culturable strains.

## 1. Introduction

The internal and external environments of the human body are inhabited by various microbial communities, including bacteria, fungi, archaea, and viruses, which collectively constitute the human microbiota [1]. Understanding the complex composition of these microbial communities is essential for characterizing the normal physiological state of human hosts and elucidating the mechanisms underlying various pathologies [2]. Numerous microbial entities inhabit different sites within the human body, such as the skin, oral cavity, and intestinal tract, establishing a symbiotic host–microbe relationship that cultivates a unique microenvironment with notable diversity.

The human microbiota remains central to microbiological research, where bacterial dynamics form the cornerstone of investigations owing to their predominance, although fungal compositions across body sites also show health correlations [3,4,5]. Firstly, the skin is the largest organ in the human body, with an average surface area of 30 m^2^ in adults [6]. Based on different microenvironments, the skin is classified as sebaceous, moist, or dry, with the scalp categorized as sebaceous [7]. The dominant phyla of microbes on the scalp are Actinobacteria, Firmicutes, Proteobacteria, and Bacteroidetes [8,9]. The primary genera identified are *Cutibacterium*, *Corynebacterium*, *Staphylococcus*, and *Streptococcus* [10]. Additionally, the human oral ecosystem is inhabited by a complex and dynamic microbial community comprising over 700 different species and 200 major types [11]. The primary microorganisms in saliva are Firmicutes, Proteobacteria, Bacteroidetes, Actinobacteria, and Fusobacteria [12]. The main genera of microorganisms in saliva are *Bacteroidetes*, *Gemella*, *Haemophilus*, *Neisseria*, *Porphyromonas*, *Prevotella*, *Streptococcus*, and *Veillonella* [13]. Lastly, the fecal microbiota is composed of over 1500 species, the main phyla of which are Firmicutes, Bacteroidetes, Actinobacteria, Proteobacteria, Fusobacteria, and Verrucomicrobia [12,14]. The most abundant genera of microorganisms in feces are *Bacteroides*, *Clostridium*, *Bifidobacterium*, *Lactobacillus*, *Bacillus*, *Enterococcus*, and *Ruminococcus* [15].

The composition of the human microbiome is closely related to health and disease [16]. Imbalances or disturbances in microbial equilibrium within the host may lead to diseases such as acne [17], ulcers [18], and diarrhea [19]. Research on the microbiological landscape of healthy populations is essential for providing baseline standards for therapeutic interventions and serves as a key element in personalized precision medicine.

In recent years, the rapid development and decreasing costs of high-throughput sequencing technologies have enhanced our understanding of the molecular composition of the human microbiome [2,20]. However, molecular diversity studies have revealed certain limitations, such as low or challenging strain resolutions [21] and potential errors introduced during the sequencing process [22]. In contrast, the composition of cultivable human microbiota still holds substantial research potential [23,24,25], particularly for understanding the human microbiome at the strain level and studying the functions of these strains [26,27]. Moreover, these methods facilitate in-depth investigations of the interactions between different human microbiota [2], the collection of probiotics and prebiotics [28], and the examination of their relationships with the host immune system [29]. Consequently, research on the human microbiome has reached a peak. However, traditional single-colony microbial culture methods face several challenges, such as labor-intensive processes and selective colony bias, potentially leading to the omission of certain cultivable microbial types. The sole reliance on high-throughput sequencing may lead to issues in detecting strains below the detection limit and those that are difficult to detect. Combining high-throughput sequencing with culture-based methods can help avoid these problems and provide a more comprehensive view of the diversity of cultivable microorganisms. This integrated approach has been effectively applied in desert environments [30]. This complementary method is expected to be an effective approach for culture-based studies of microbial community structures.

For large-scale analyses of the human microbiome, samples collected from the field must be transported to the laboratory for preservation before subsequent cultivation studies. Therefore, pre-treatment transport conditions are crucial. Previous studies have mainly focused on the effects of transportation and storage methods on the molecular composition of the human microbiome [31,32]. However, their effects on culturable microorganisms, particularly in non-fecal samples, have received less attention [33]. The effectiveness of rapid cooling with liquid nitrogen as a pre-treatment method, which potentially protects cellular structures by forming large ice crystals within the cells, remains to be confirmed [34]. Transportation temperature, controlled by ambient temperature or external media such as ice packs and dry ice [35], can influence the composition of microbial communities. Additionally, transport buffers, such as glycerol and dimethyl sulfoxide (DMSO), play positive roles in maintaining cell viability [33,36]. However, the effects of liquid nitrogen treatment, transport temperature (room temperature, ice packs, and dry ice), and transport buffers (DMSO and glycerol) on culturable human microorganisms remain unclear and require further investigation.

**Figure 1:**
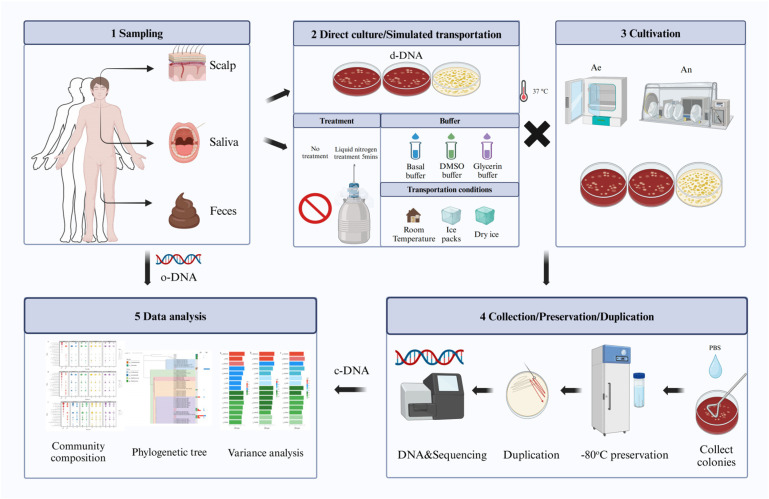
Framework of the experimental design. 1—Sampling: Samples were collected from three body sites (scalp, saliva, and feces) of three volunteers. 2—Direct Culture: The samples were cultured on two types of media: Columbia blood agar plates for scalp and saliva samples and Gifu anaerobic broth (GAM) medium plates for fecal samples (direct-culture samples of DNA and d-DNA). Some samples underwent simulated transport processing and were divided into liquid nitrogen treatment (Treatment) and untreated (No Treatment) groups. Different buffers were added, including the basal buffer, 5% DMSO buffer, and 20% glycerol buffer. Simulated transport conditions included room temperature, ice packs, and dry ice. 3—Cultivation: Samples were incubated under aerobic (Ae) and anaerobic (An) conditions at 37 °C. 4—Collection/Preservation/Duplication: Colonies were collected by adding phosphate-buffered saline (PBS) and scraping them from the plates. To preserve the samples, they were added to glycerol buffer to achieve a final concentration of 20% and stored at −80 °C. Some samples were re-cultured by streaking and isolating single colonies for further culture. DNA extraction and sequencing, including Illumina sequencing and full-length 16S rRNA gene Sanger sequencing of single colonies, were performed. DNA extracted from cultured samples was termed culturable samples’ DNA (c-DNA). 5—Data Analysis: The primary analyses included comparing the diversity of DNA extracted from original samples denoted as original samples’ DNA (o-DNA) and culturable samples’ DNA (c-DNA), analyzing the phylogenetic trees of re-cultured preserved colony mixtures, and assessing differences under various simulated transport conditions. Images were generated using BioRender.com.

This study aimed to explore the composition of culturable microorganisms in the human microbiome by integrating culturable and molecular ecological methods. Additionally, we investigated the effect of different transportation methods on the cultivable microbiome of the human body by considering the community composition characteristics of the microorganisms (Figure 1). Through this study, we hope to gain a better understanding of cultivable microorganisms in the human microbiome and optimize transportation conditions to enhance the cultivation efficiency of these microbes. This will provide valuable references for subsequent strain-level studies and the discovery of novel human-associated microorganisms.

## 2. Materials and Methods

### 2.1. Recruitment and Sample Collection

Three healthy volunteers with an average age of 25.7 ± 4.6 years (mean ± SD, two females and one male, sedentary students, omnivorous) were recruited from Fudan University in Shanghai. None of the patients had taken antibiotics or related medications in the past two weeks. This study was approved by the Ethics Committee of Fudan University (FE23022R).

Scalp, saliva, and fecal samples were collected by the participants on their own. In the morning, fecal samples were collected during the volunteers’ first bowel movement, followed by scalp and saliva samples. The scalp was wiped with moist disposable sterile iCleanhcy Specimen Collection Swabs (CY-98000; HCY Technology, Shenzhen, China) soaked in SCF-1 (Sample Collection Fluid) solution (50 mM Tris buffer, pH 7.6; 1 mM EDTA, pH 8.0; and 0.5% Tween-20) for 30 s each on the left and right sides (2 cm × 2 cm areas) [37]. Saliva samples (2 mL) were collected in funnel-type screw-cap microtubes (B80180; BOOPU Biotechnology, Changzhou, China). Fecal samples were collected using a 5 mL feces collector with a spoon (BIORISE, Shanghai Longfu Industrial Co., Ltd., Shanghai, China).

### 2.2. Simulating Transportation and Cultivation

The samples collected on the same day were divided into two groups. One group was treated with liquid nitrogen, whereas the other group was left untreated, and a transport protection buffer was subsequently added. Scalp swabs were immersed in 5 mL of SCF-1 solution, thoroughly mixed, and divided into six aliquots. Three aliquots were treated as follows: one without transport protection buffer, one with 20% glycerol as transport protection buffer, and one with 5% DMSO as transport protection buffer. Saliva samples were collected in 1 mL volumes, mixed with 4 mL of SCF-1 buffer, and treated with the same transport protection buffers as the scalp samples. Fecal samples were collected by placing 1 g of the sample in a collection tube containing 5 mL of CB solution (HaiBo Co., Qingdao, China) [38,39], thoroughly mixed, and processed similar to the other sample types. The liquid nitrogen treatment involved placing the sample in a liquid nitrogen solution for 5 mins.

Five copies of each sample were made in duplicate; one copy was placed at −80 °C to extract the original samples’ DNA, and one copy was applied directly to a Colombian blood (5% sheep blood) plate (Nanjing Quansheng Biotechnology Co., Ltd., Nanjing, China; Supplementary Table S1) or GAM medium plate (HaiBo Co., Qingdao, China; Supplementary Table S1) to serve as a standard for direct-culture samples’ DNA. The remaining three samples were transported for 2 days under three simulated environmental conditions: room temperature, ice packs, and dry ice. Subsequently, aerobic cultivation was performed at 37 °C in a constant-temperature incubator using nutrient medium for 2 days. Additionally, one set of cultures was incubated anaerobically at 37 °C for 3 days. Columbia blood agar (5% sheep blood) was used as the nutrient medium for scalp and saliva samples [9], and GAM was used for fecal samples [40,41]. All anaerobic media were treated in advance by overnight incubation in an anaerobic chamber. After 2–3 days, 1 mL of PBS solution was added to the Petri dishes, and the colonies were harvested using a sterile swab and transferred to a 2 mL sterile plastic tube. The samples were thoroughly mixed and supplemented with 20% glycerol buffer for storage at −80 °C. This procedure was used for subsequent DNA extraction to determine the composition of cultivable bacterial communities in the human body. Microbial colony counts were performed on culture plates at three sites of the human body. Aerobic plate count methods were employed for scalp and saliva samples, and anaerobic plate count methods were used for fecal samples.

### 2.3. DNA Extraction, Library Preparation, and Sequencing

Extraction of the original DNA samples (o-DNA, three replicates at three sites, yielding nine total samples) was performed using the DNaeasy PowerSoil Pro kit (QIAGEN, Venlo, The Netherlands). Based on the manufacturer’s instructions, slight optimization was performed by treating the samples with lysozyme (10 mg/mL, 37 °C) for 2 h before cell lysis by 180 rpm shaking. All other procedures were performed according to the instructions of the manufacturers. DNA from culturable samples (c-DNA) under aerobic and anaerobic conditions was extracted using an Axygen DNA Extraction Kit (Axygen, Union City, CA, USA) according to the manufacturer’s instructions, with minor modifications (Proteinase K treatment extended to 2 h; Sangon Biotech Co., Ltd.). The culturable samples were divided into 18 samples for direct-culture DNA samples (d-DNA, three replicates at three sites under two oxygen conditions) and 324 samples after simulated transportation (same sampling conditions under 18 simulated transport conditions). A total of 351 samples were used for DNA sequencing (9 for o-DNA, 18 for d-DNA, and 324 for c-DNA). It is worth emphasizing that the DNaeasy PowerSoil Pro kit has the advantage of extracting a low abundance of microorganisms from the original samples. Culturable samples have high microbial counts, and the Axygen DNA extraction kit can fulfill experimental needs [30].

The universal primer set 21F/338R for the V1-V2 regions of the bacterial 16S rRNA gene with 12 nt barcodes for scalp and saliva samples, which are useful for differentiating microorganisms in skin and oral community studies, was used [42,43]. The bacterial V4 region primer set 515F/806R with 12 nt barcodes was used to analyze fecal samples, which have a higher resolution [44,45]. PCR reactions for each sample were performed with a total volume of 25 μL of 0.2 μM forward primer, 0.2 μM reverse primer, 10 ng of template DNA, and 12.5 μL of 2 × Ex Taq dNTP Mixture (Takara Biotechnology Co., Ltd., Dalian, China). The reaction conditions were as follows: 95 °C for 5 mins, followed by 30 cycles of 95 °C for 30 s, 55 °C for 30 s, and 72 °C for 60 s, with a final extension at 72 °C for 10 mins; then, it was held at 4 °C. The DNA concentration of the PCR products was measured using Qubit 4 (Thermo Fisher Scientific Inc., Waltham, MA, USA). Library preparation with 100 ng of DNA input was performed using the VAHTS Universal DNA Library Prep Kit for Illumina V3 (Vazyme International LLC, Nanjing, China) and VAHTS clean DNA beads (Vazyme International LLC, Nanjing, China), according to the manufacturer’s instructions. The amplicon libraries were sequenced on an Illumina NovaSeq PE250 platform (Illumina, San Diego, CA, USA) at Shanghai Paisano Biotechnology Co., Ltd. (Shanghai, China). QC cassette sequencing was performed according to the manufacturer’s instructions.

### 2.4. Isolating and Culturing Single Strains

Five samples were selected from culturable samples, including one each from aerobic and anaerobic culturable scalp and saliva samples, as well as one from anaerobic culturable samples of feces, which were preserved at −80 °C. Cultures were grown under the same conditions as described above, and the stock solution was diluted in PBS. Fifteen to twenty individual colonies were selected for each culturable sample, and the full-length 16S rRNA gene was amplified using the bacterial primer set 27F/1492R [46]. PCR was performed directly using colonies as templates in the previous conditions, except for the extension of the initial temperature of 95 °C to 10 mins. Sanger sequencing with paired-end reads was performed by Sangon Biotech Co., Ltd. (Shanghai, China).

### 2.5. Data Analyses

The analysis workflow for high-throughput sequencing data was as follows: Raw sequencing data were analyzed using the qiime2 pipeline [47], which included merging paired-end data and demultiplexing samples based on barcodes, quality control, and filtering. Subsequently, the optimized data were processed using the DADA2 denoising method to obtain representative Amplicon Sequence Variants (ASVs), abundance information tables, and taxonomic classification information for representative sequences using the Silva-138 database [48]. Rarefaction curves and diversity metrics (ASV, Shannon, Inverse Simpson, Pielou, and PD_whole_tree metrics) of the samples were calculated and visualized using R software (version 4.3.3), and the differences between groups were tested using the two-sample Student’s *t*-test.

Beta diversity analysis was mainly based on Bray–Curtis dissimilarity to calculate the compositional differences between samples. Principal coordinate analysis (PCoA) was used to determine the differences in bacterial 16S rRNA genes at the three sites in the human body. Similarity-based analysis (ANOSIM) was used to examine the differences in the communities of the three sites in the human body, as well as the culturable samples under different transportation conditions. A PERMANOVA method based on the Bray–Curtis dissimilarity matrix was used to reveal the factors (liquid nitrogen treatment, transport environment, and transport buffers) that influence the composition of bacterial communities in the three human body sites under different transportation conditions, using the adonis2 function in the “vegan” package.

LEfSe analysis was used to identify microbial markers with statistically significant differences between groups using different transportation methods [49]. A phylogenetic tree was constructed using ClustalW for alignment, MEGA11 [50] for Maximum Likelihood analysis using the Tamura–Nei model and 1000 bootstrap replicates, and iTOL v6.9 (https://itol.embl.de, accessed on 27 July 2024) for visualization. Full-length 16S rRNA gene sequences from bidirectional sequencing were aligned and assembled using the SeqMan software (DNAstar, v7.1.0). Sequencing results were annotated using the EzBioCloud 16S rRNA gene database [51]. Sequence alignment of the 16S rRNA gene was performed using ClustalW, and phylogenetic tree analysis and visualization were performed using the same methods used for the ASV analysis.

## 3. Results

### 3.1. Comparison of Original and Culturable Microorganisms of the Human Microbiome

Three volunteers were recruited, and samples were collected from the scalp, saliva, and feces. High-throughput sequencing of the bacterial composition was performed on the original microorganisms and culturable microorganisms. A total of 310 (88%) 16S rRNA gene sequencing samples were obtained for subsequent analyses (98 scalp, 107 saliva, and 105 feces samples). Regions V1–V2 of the 16S rRNA gene were selected for scalp and saliva samples, whereas region V4 was selected for fecal samples. After barcode identification, quality control, denoising, and other filters, 6,926,412 high-quality reads were obtained (1,053,056 for the scalp, 1,219,355 for saliva, and 4,654,001 for feces). Denoising using DADA2 yielded 5694 ASVs (1557 for scalp, 2267 for saliva, and 2130 for feces). ASVs representing >1% of the samples were retained. After data cleaning, 579 ASVs were obtained from three sites (214 for the scalp, 308 for saliva, and 138 for feces) (Table 1, Supplementary Figure S1). These data were used for subsequent analyses. A total of 117 ASVs from the original DNA samples and 518 ASVs from the culturable DNA samples were obtained by sequencing the three human body sites. In the culturable bacterial composition, the sample with the most ASVs was the saliva, followed by the feces and scalp samples.

**Figure 2:**
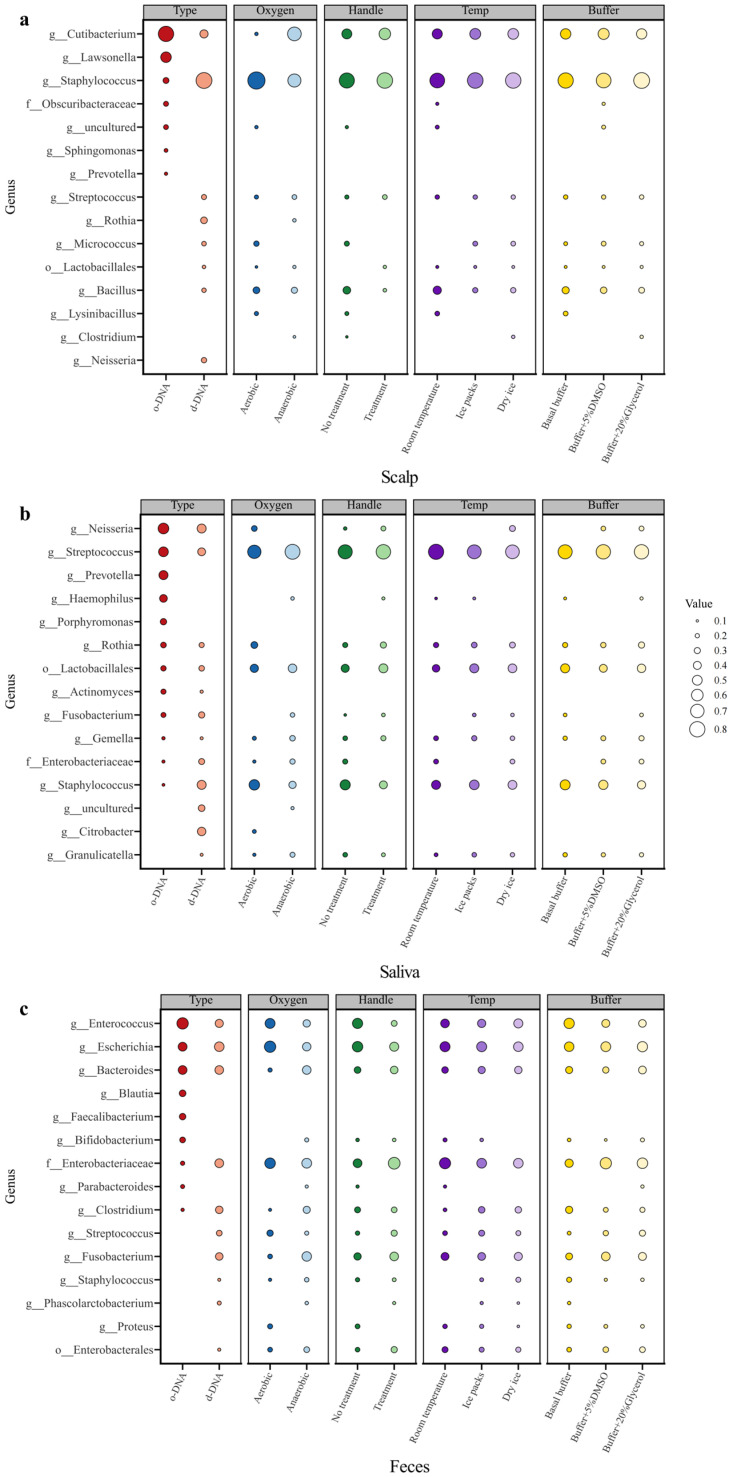
Bubble diagrams of the top 15 genera of original and culturable microorganisms from three sites of the human body: (**a**)—scalp; (**b**)—saliva; (**c**)—feces. Type represents a comparison between the original samples’ DNA and direct-culture samples’ DNA. Oxygen represents a comparison between microorganisms that can be cultured under aerobic and anaerobic conditions. Handle represents a comparison between treatment without liquid nitrogen and treatment with liquid nitrogen. Temp represents a comparison between simulated ambient transport temperatures: room temperature, ice packs, and dry ice. Buffer represents a comparison between basal buffer, 5% DMSO buffer, and 20% glycerol buffer. Original samples’ DNA is indicated by o-DNA. Culturable samples’ DNA is indicated by c-DNA.

The bacterial alpha diversity nearly reached saturation, indicating that the sequencing depth for both the original and culturable microorganisms was adequate (Supplementary Figure S1). The main phyla of the culturable microorganisms on the scalp were Firmicutes, Actinobacteria, and Proteobacteria (Supplementary Figure S2), while the main genera identified were *Staphylococcus*, *Cutibacterium*, *Bacillus*, *Streptococcus*, and *Micrococcus* (Figure 2, Supplementary Figure S3, Supplementary Table S2). The main genera that grew under aerobic conditions were *Staphylococcus* and *Bacillus*, whereas those that grew under anaerobic conditions were mainly *Cutibacterium*.

The main culturable microorganisms in saliva were Firmicutes, Proteobacteria, and Actinobacteria (Supplementary Figure S2), while the main genera were *Streptococcus*, *Staphylococcus*, *Rothia*, *Neisseria*, *Granulicatella*, and *Gemella* (Figure 2, Supplementary Figure S3, Supplementary Table S3). *Streptococcus* and *Gemella* are facultative anaerobic bacteria that can grow under both aerobic and anaerobic conditions. *Neisseria* and *Rothia* are primarily found under aerobic conditions. *Haemophilus* and *Granulicatella* accounted for a certain percentage of the culturable microorganisms. These two genera are nutritionally deficient, but other microorganisms in the medium (e.g., *Staphylococcus*) can provide the required nutrients, fully demonstrating the advantages of culturomics.

The main culturable phyla in the feces were Proteobacteria, Firmicutes, Fusobacteria, and Bacteroidetes (Supplementary Figure S2), while the main genera were *Enterococcus*, *Escherichia*, *Bacteroides*, *Bifidobacterium*, *Parabacteroides*, and *Clostridium* (Figure 2, Supplementary Figure S3, Supplementary Table S4). *Bacteroides*, *Bifidobacterium*, *Parabacteroides*, and *Clostridium* were the main genera of culturable bacteria under anaerobic conditions. *Enterococcus*, *Escherichia* facultative anaerobes, accounted for a high percentage under both aerobic and anaerobic conditions. Although *Fusobacterium* had a low percentage in the original samples, there was a large increase in its percentage in the culturable group. *Staphylococcus* and *Streptococcus* had a moderate percentage in the fecal culturable group of samples, but the ASVs were of a different type than those from the scalp and oral cavity (Supplementary Tables S5 and S6).

**Figure 3:**
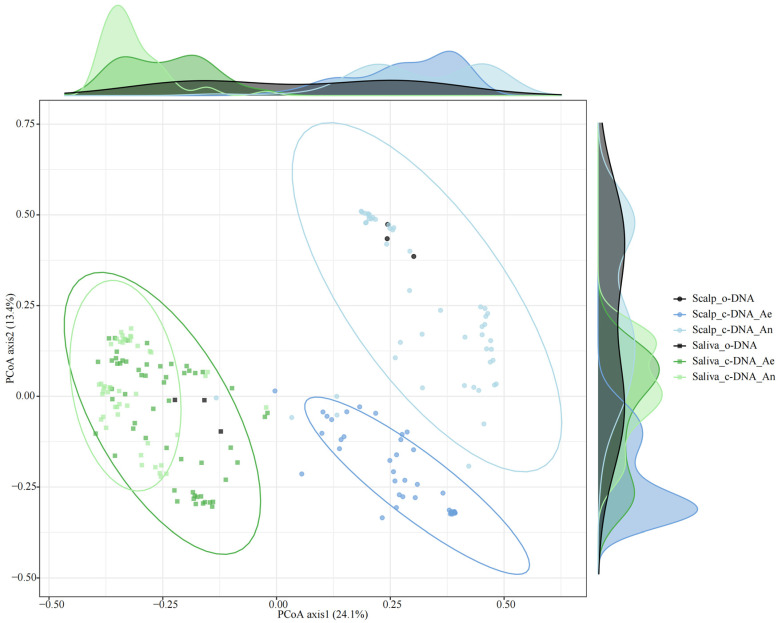
PCoA plots based on Bray–Curtis dissimilarity showing the differences between original and culturable microorganisms at two sites (scalp and saliva) of the human body under aerobic and anaerobic conditions. Original samples’ DNA is indicated by o-DNA. Culturable samples’ DNA is indicated by c-DNA. Aerobic and anaerobic conditions are indicated by Ae/An. Circles represent 95% confidence intervals for different c-DNA samples.

The representative culturable bacteria from different body sites exhibited distinct differences (Figure 3 and Supplementary Figure S4). At the genus level, culturable microorganisms accounted for more than 60% of the original samples from the three different sites in the body (scalp, 64%; saliva, 63%; and feces, 65%) (Supplementary Tables S2–S4). Overall, the cultivation methods employed successfully yielded the predominant microbial types from the three distinct human body sites.

### 3.2. Bacterial Diversity Between Original and Culturable Microorganisms of the Human Microbiome

**Figure 4:**
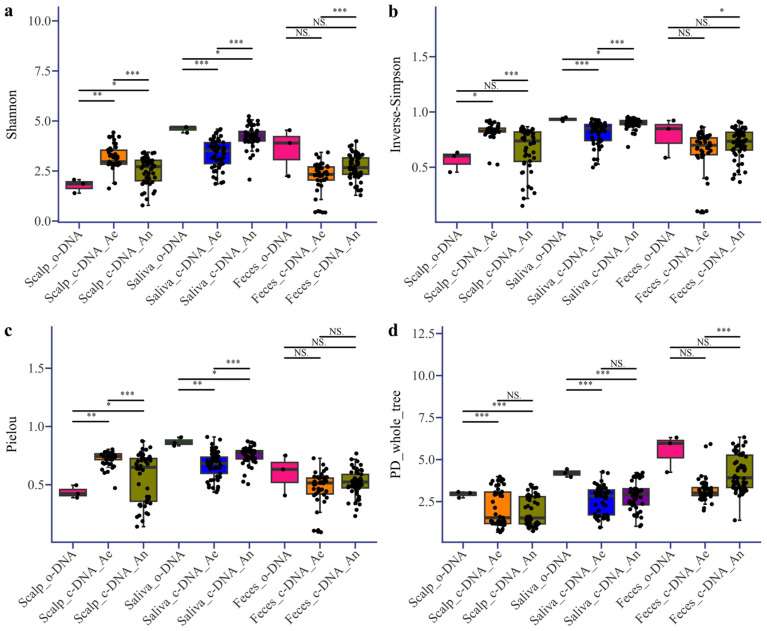
Alpha diversity indices of microorganisms from three human body sites (scalp, saliva, and feces) before and after cultivation under aerobic and anaerobic conditions. (**a**)—Shannon, (**b**)—Inverse Simpson, (**c**)—Pielou, and (**d**)—PD_whole_tree. (Original samples’ DNA is indicated by o-DNA. Culturable samples’ DNA is indicated by c-DNA. Aerobic and anaerobic conditions are indicated by Ae/An. The *t*-test was used, and significance levels are denoted as follows: NS (not significant), * *p* < 0.05, ** *p* < 0.01, *** *p* < 0.001.)

PCoA revealed distinct bacterial profiles at the three sites in the human body (Figure 3, Supplementary Figure S4). Alpha diversity analysis revealed that after culturing, the alpha diversity of the scalp improved and even exceeded that of the original samples, indicating that some rare skin strains were cultivated. In contrast, the alpha diversity of the saliva and fecal samples after culture was still lower than that of the original samples (Figure 4a–c). However, considering the evolutionary relationships (Figure 4d), the diversity of PD_whole_tree after culture was still lower than that of the original samples, suggesting that culturomics can improve the recovery of strains with closer evolutionary relationships. The level of alpha diversity after culturing the saliva samples was higher than that of the scalp and feces samples. However, when considering evolutionary relationships, alpha diversity before and after the fecal culture was the highest (Figure 4d). Furthermore, the bacterial diversity after the scalp culture under aerobic conditions was higher than that under anaerobic conditions, whereas the opposite was true for saliva and feces. This also reflects the oxygen preference of bacteria at different sites.

### 3.3. Recultivation of Major Cultivable Microorganisms from the Human Microbiome

Analysis of bacterial diversity at three human body sites (scalp, saliva, and feces) through culture-based methods not only revealed the types of cultivatable microorganisms but also reflected their composition. After analyzing the diversity of cultivatable microorganisms, individual colonies could be isolated and preserved by plating and single-colony selection from cultures stored at −80 °C. Full-length 16S rRNA gene sequencing was performed on five samples from three selected sites. The results showed that the culture-based method involving high-throughput sequencing of all bacteria scraped from the plates contained over 60% of the bacterial genera from the three human body sites (scalp, saliva, and feces).

**Figure 5:**
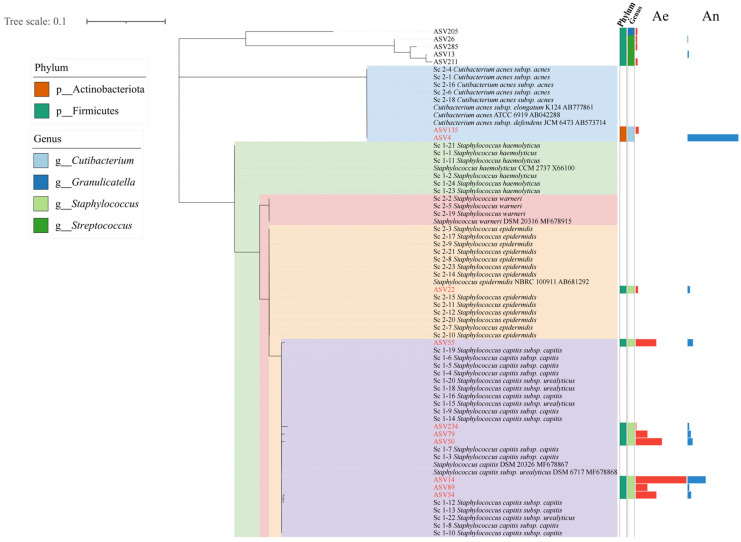
A phylogenetic tree of the 16S rRNA gene constructed from the full-length sequencing results of single strains isolated from scalp cultivation samples and the major ASVs (amplicon sequence variants) obtained through high-throughput sequencing. Phyla and genera are shown on the left. The columns labeled Ae and An represent the distribution of selected major ASVs’ relative abundances in aerobic and anaerobic cultures, respectively. Labels beginning with Sc indicate the closest annotated sequence for the selected single colony from the scalp sample. ASVs labeled in red font represent the ASV sequence is matched with 16S rRNA gene sequence of one of recultured single colony bacteria.

A 16S rRNA gene phylogenetic tree constructed using both high-throughput sequencing-derived ASVs and full-length sequences from three human body-site cultures (Figure 5 and Supplementary Figures S5 and S6) revealed concordance between the isolated bacterial strains and their corresponding ASVs. Specifically, the scalp samples contained diverse *Staphylococcus* strains, including two subspecies of *Staphylococcus capitis* (*Staphylococcus capitis* subsp. capitis and *Staphylococcus capitis* subsp. urealyticus), as well as *Staphylococcus epidermidis*, *Staphylococcus warneri*, and *Staphylococcus haemolyticus*. Additionally, the proportion of *Cutibacterium acnes* significantly increased under anaerobic conditions (Figure 5). In the saliva samples, *Streptococcus parasanguinis* and *Streptococcus gordonii* emerged as the predominant *Streptococcus* species. Interestingly, a *Staphylococcus* strain (*Staphylococcus argenteus*) distinct from the typical salivary microbiota was identified, highlighting the spatial heterogeneity of the *Staphylococcus* strain distribution across different body sites (Supplementary Figure S5, Supplementary Table S5). Fecal samples yielded isolates primarily belonging to highly abundant genera, including *Bacteroides* (*Bacteroides thetaiotaomicron* and *Bacteroides fragilis*), *Escherichia* (*Escherichia fergusonii* and *Shigella flexneri*), and *Enterococcus* (*Enterococcus gallinarum, Enterococcus lactis*, and *Enterococcus faecalis*) (Supplementary Figure S6).

### 3.4. Effects of Different Transportation Conditions on Culturable Human Microorganisms

Different transportation conditions significantly influenced the subsequent culture of microorganisms. In this study, the order of magnitude of their effects on the bacterial composition was as follows: liquid nitrogen treatment, transportation temperature, and different buffers (Table 2). Multiple permutation analysis (PERMANOVA) revealed that the sampling site (scalp, saliva, and feces), source (different volunteers), and liquid nitrogen treatment had significant (*p* < 0.001) effects on the type of bacteria cultivated, accounting for 30%, 8%, and 2% of the total variance, respectively.

**Figure 6:**
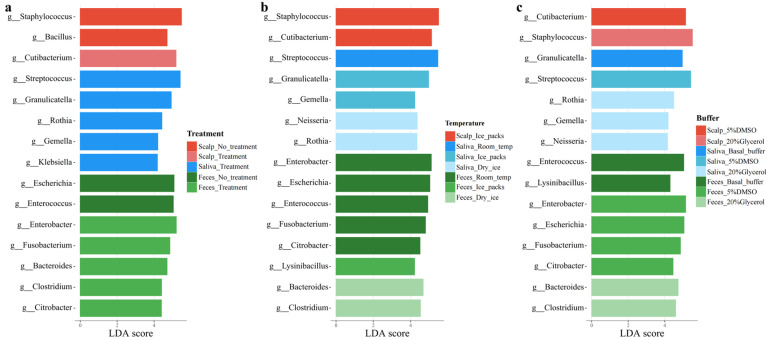
Histogram of LEfSe-based analysis of liquid nitrogen treatment (**a**), transportation temperature (**b**), and buffers (**c**) on culturable microorganisms at three sites. Linear discriminant analysis (LDA) showed genera with significant differences.

Based on linear discriminant analysis (LEfSe), we further investigated the effects of three different transportation conditions (liquid nitrogen treatment, transportation temperature, and buffers) on cultivated bacterial types and found that different transportation temperatures had significant effects on the composition and diversity of the bacteria (Figure 6).

As shown in Figure 6a, liquid nitrogen treatment increased the number of microorganisms at different sites, especially in the saliva and fecal samples, where multiple genera were significantly enhanced. Simultaneously, liquid nitrogen treatment reduced the percentage of *Bacillus* in the scalp samples, which may reduce the competition of *Bacillus* for the growth resources of other types of microorganisms. However, liquid nitrogen treatment also reduced the percentage of *Staphylococcus* in scalp samples and should be avoided if staphylococcal growth is a concern. Regarding the transportation temperature (Figure 6b), dry ice transportation was the best choice. For example, dry ice transport in the scalp showed a higher proportion of *Staphylococcus* (Supplementary Figure S7), saliva had significantly higher percentages of *Neisseria* and *Rothia*, and feces had significantly higher percentages of *Bacteroidetes* and *Clostridium*. For buffers, the DMSO buffer increased the percentage of *Cutibacterium* in the scalp, *Streptococcus* in the saliva, and *Enterobacter* and *Citrobacter* in the feces more significantly. Glycerol buffer increased the percentage of *Staphylococcus* in the scalp; the percentages of *Rothia*, *Gemella*, and *Neisseria* in the saliva; and the percentages of *Bacteroides* and *Clostridium* in the feces. However, in combination with the colony counts of culturable microorganisms from the three human body sites (Supplementary Tables S7–S9), liquid nitrogen treatment, dry ice transport, and DMSO buffer proved to be the optimal solutions for simulating the transport conditions of human microbiome cultures. These optimized transport conditions effectively preserved the integrity and diversity of the microbial samples, providing a basis for subsequent microbial cultures and research.

## 4. Discussion

This study focused on culturable microorganisms in human scalp, saliva, and fecal samples; described the bacterial species in these three types of samples; and found that the core bacterial diversity in the original samples could be effectively cultured (Figure 2, Table 1 and Supplementary Tables S2–S4) based on a combination of culturomics and high-throughput sequencing approaches. This method can capture numerous low-abundance microorganisms from the original samples, thereby culturing rare species that may be crucial for regulating the metabolic functions of local microbial communities [52]. Moreover, co-culture models using cultivation-based methods can simultaneously address the interactions between different microbial strains. For example, nutritionally deficient *Haemophilus* and *Granulicatella* were cultured from saliva samples (Supplementary Table S3) [53]. Additionally, for microorganisms that cannot be isolated using single-colony methods, culturomics can be used to increase the proportion of target strains in the culture, thereby facilitating subsequent single-strain separation. This approach can enhance the effectiveness of microbial isolation and identification processes [24,54].

In the analysis of microbiomes across different body sites, similar to the results of molecular diversity analysis [12], significant differences were observed in cultivable bacteria among various human body sites (Figure 2). From a physiological perspective, the types of bacteria cultured under aerobic and anaerobic conditions on the scalp differed significantly, whereas those cultured in saliva and feces showed less variation [1]. This phenomenon was also reflected in the alpha diversity analysis (Figure 4), which revealed significant differences in the physiological characteristics of the skin, oral cavity, and gut. The skin, exposed to the external environment, primarily harbors aerobic-preferring microorganisms, such as *Staphylococcus*, whereas in a relatively enclosed hair follicle environment, *Cutibacterium* is more common [55]. Anaerobic microorganisms are advantageous in the relatively enclosed environments of the oral cavity and gut [56,57]. Additionally, the diversity of culturable microorganisms on the skin was higher than that of the original samples, with *Staphylococcus* being the most prevalent genus among the culturable microorganisms. Combined with the results of single-colony re-culture, *Staphylococcus* on the scalp was characterized by diverse strains (Figure 5). These findings provide strong evidence for strain-level diversity studies of *Staphylococcus* on the skin and support further functional and physiological research on single-colony candidates [58,59]. This supports the idea that the type of *Staphylococcus* differs in different parts of the body [60,61]. In feces, *Bifidobacterium* and *Bacteroides* can be cultured (Figure 2c), suggesting that they have the potential to be used as probiotics to restore healthy gut microbiota [62,63]. These findings provide a solid foundation for describing culturable microorganisms in individuals and facilitate the large-scale exploration of the human microbiome. Based on the combination of culturomics and high-throughput sequencing, this method can efficiently obtain richer microbial taxa than by directly picking single colonies and can cover a higher degree of culturable microorganisms.

Overall, more than 60% of the major bacterial genera were identified from the three body sites using culturomics (Figure 2, Supplementary Tables S2–S4). During cultivation, the predominant bacterial types in the samples, particularly various species of *Staphylococcus*, were identified (Figure 5). The cultivation of these microorganisms provides opportunities for future strain-level investigations, enabling the analysis of strain-specific variations. Furthermore, this integrated approach, which combines first- and second-generation sequencing methodologies, offers an efficient strategy for identifying novel bacterial strains. More importantly, diversity analyses based on culturable microorganisms can reflect the types of viable microorganisms and their physiological characteristics. Compared to DNA-based molecular ecological analysis, this approach can more effectively reveal the composition of active strains and functional contributor communities in the original niches, as well as the core active human microbiota [64]. In the future, the integration of culturomic and single-cell isolation approaches for microbial analysis will facilitate in-depth investigations into strain-level diversity, dynamic functional variations, and host–microbe interactions of the same bacterial species across different body sites.

Regarding transport conditions, PERMANOVA indicated that liquid nitrogen treatment significantly improved the abundance of major microbes and had a substantial impact on microorganisms from different sites in the human body. This was mainly reflected in the increase in the proportion of microorganisms of several genera and the richness of culturable species, which facilitated the recovery of the community composition of the original samples [65]. Notably, this treatment may not be suitable for the culture of *Staphylococcus* on the scalp. Therefore, the application of liquid nitrogen treatment at different sites in the human body must be tailored according to the specific microbial types at each site. Although the differences in transportation temperature and buffer solutions were not significant in the PERMANOVA, LEfSe results indicated that dry ice transport had a significant advantage in maintaining the main cultivable bacteria and diversity. This phenomenon may be attributed to reduced microbial metabolic activity at low temperatures, such as the inhibition of *Bacillus* growth, which lowers the risk of contamination and consequently has a positive impact on restoring the community composition of the original samples [66]. A combination of differential diversity analysis and colony count results indicated that the DMSO buffer demonstrated superior performance. Biclot et al. suggested that adding a DMSO buffer solution to fecal samples is preferable, which aligns with the findings of this study [33]. Notably, *Enterobacter* (21% of the bacteria in culturable fecal samples) and *Citrobacter* (4% of the bacteria in culturable fecal samples) showed significant increases in culturable microbial samples in which DMSO buffer was used as transportation condition, even though both were not the predominant microbial types in the feces original samples. This suggests that the DMSO buffer has certain advantages in enhancing the diversity of low-abundance culturable bacterial species in fecal samples.

This study has certain limitations. Although this experiment involved the sampling of three volunteers, the results showed large variation among participants. Therefore, data from other volunteers need to be further analyzed for in-depth study and validation of the experimental results [67]. Owing to the highly diverse nature and varying growth conditions of human bacteria, future research should optimize the experimental design by exploring the effects of different culture media types [24]. Additionally, this study focused solely on bacterial types and did not address the cultivation of fungi and archaea [68].

## 5. Conclusions

Combining culturomics with single-colony isolation methods not only enhances the precision of human microbiome research at the strain level but also improves efficiency and specificity while reducing labor intensity. This approach has led to significantly advanced studies on human microbial strain diversity, interspecies interactions, and host–microbe interactions. During the research process, the optimization of transportation conditions in the preliminary stages ensured the acquisition of maximum microbial diversity from the samples, providing critical support for subsequent experimental analyses. These advancements not only offer novel perspectives for understanding the human microbiome but also open up new avenues for future applications of microbiome research. Through these studies, we can better comprehend the relationship between microorganisms and human health and further explore their potential roles in disease prevention and treatment.

## Figures and Tables

**Table 1:** Number of ASVs for original DNA samples and culturable DNA samples at the three human body sites.

Site	Original Samples’ DNA ASVs	Direct-Culture Samples’ DNA ASVs	Culturable Samples’ DNA ASVs ^a^	Both ASVs ^b^
Scalp	17 ± 5	33 ± 8	29 ± 19	12 ± 5
Saliva	40 ± 4	22 ± 9	47 ± 22	28 ± 4
Feces	61 ± 14	54 ± 14	34 ± 13	55 ± 18

^a^ Culturable sample DNA results include DNA from direct-culture samples (d-DNA) and cultures following simulated transport. ^b^ Both ASVs represent ASVs obtained from both original samples’ DNA and culturable samples’ DNA. All results are based on three-volunteer calculations of means and standard deviations.

**Table 2:** Effect of different transportation conditions on human culturable microorganisms based on Bray–Curtis differential PERMANOVA analysis.

Factors	Df	Sum of Sqs	R^2^	F	Pr (>F)	Sig.
Site	2	39.916	0.30331	76.395	0.001	***
Source	2	10.33	0.07849	19.7698	0.001	***
Liquid nitrogen treatment	1	2.354	0.01789	3.0033	0.001	***
Transportation temperature	2	0.598	0.00455	1.1449	0.275	
Buffers	2	0.551	0.00418	1.054	0.37	
Residual	298	77.853	0.59158			
Total	309	131.602	1			

Sig. indicates significance; *** indicates *p* < 0.001.

## Data Availability

The sequencing data from this study are available in the National Omics Data Encyclopedia (https://www.biosino.org) repository under accession number OEP005479 (https://www.biosino.org/node/project/detail/OEP005479, accessed on 29 August 2024).

## Supplementary Materials

The following supporting information can be downloaded at https://www.mdpi.com/article/10.3390/microorganisms13030549/s1. The appendix includes information on Supplementary Figures S1–S7 in Supplementary Information.docx. Supplementary Figure S1: Rarefaction curves for each sample of human ASVs from the original and culturable samples’ DNA; Supplementary Figure S2: Phylogenetic trees comparing human microbiomes based on ASVs from the original and culturable microorganisms; Supplementary Figure S3: Histograms of the top 20 genera found in the original and cultured samples’ DNA; Supplementary Figure S4: PCoA plots based on Bray-Curtis dissimilarity showing the differences between original and culturable microorganisms at one site (feces) in the human body under aerobic and anaerobic conditions; Supplementary Figure S5: Phylogenetic tree of the 16S rRNA gene constructed from the sequencing results of single strains isolated from saliva samples and major ASVs obtained through high-throughput sequencing; Supplementary Figure S6: Phylogenetic tree of the 16S rRNA gene constructed from the sequencing results of single strains isolated from feces cultivation samples and major ASVs obtained through high-throughput sequencing; Supplementary Figure S7: Heatmap of the correlation analysis between transport temperature and culturable microorganisms from human scalp based on ASV-level analysis. Supplementary Table S1 shows composition of medium; Supplementary Tables S2–S4 show top 15 genera of scalp, saliva, and feces, respectively; Supplementary Tables S5 and S6 shows proportions of ASVs in *Staphylococcus* and *Streptococcus*, respectively; Supplementary Tables S7–S9 show colony counts of cultivable microorganisms from scalp, saliva, and feces samples, respectively, in Supplementary Table.xlsx.

## Abbreviations

ASVs	Amplicon Sequence Variants
NS	Not significant
PBS	Phosphate-buffered saline
PCoA	Principal coordinate analysis
