# Supplementary material for: Culturable Human Microorganisms and the Impact of Transportation Conditions on Cultivability
Source: Microorganisms. 2025 Feb 28;13(3):549. doi: 10.3390/microorganisms13030549 (PMC11944332; doi:10.3390/microorganisms13030549)
Supplement: Supplementary file 1 [file microorganisms-13-00549-s001.zip › Supplementary_Information_0206(4)-qzx.pdf]

Supplementary Information

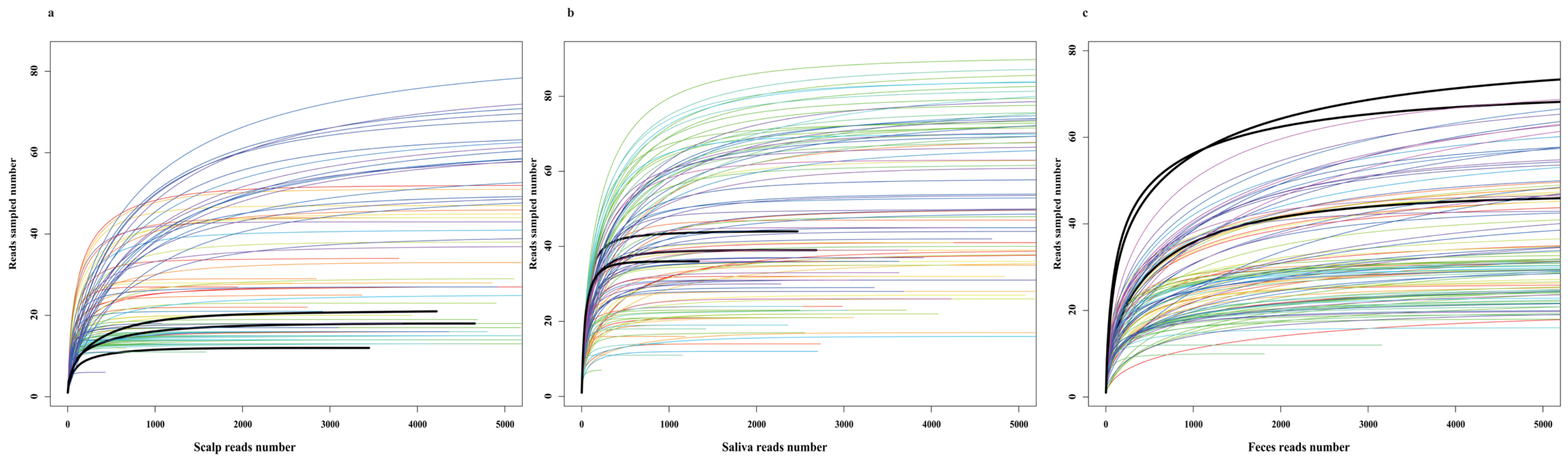

Supplementary Figure S1. Rarefaction curves for each sample of human ASVs from the original and culturable samples' DNA (original samples' DNA is represented by black lines, and culturable sample DNA is represented by colored lines). a) Sequencing depth and sequencing depth of each scalp sample, b) sequencing depth and sequencing depth of each saliva sample, and c) sequencing depth and sequencing depth of each fecal sample.

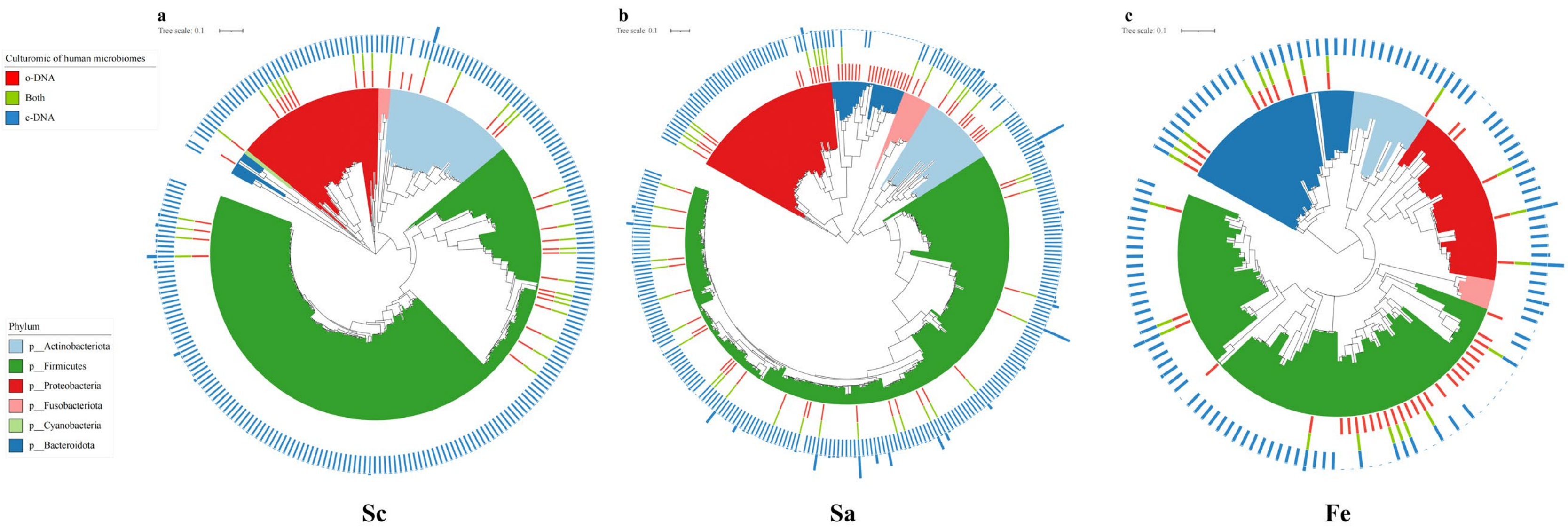

Supplementary Figure S2. Phylogenetic trees comparing human microbiomes based on ASVs from the original and culturable microorganisms. a) Phylogenetic tree constructed from ASVs representing > 1% of the scalp (Sc) microbiome, b) phylogenetic tree constructed from ASVs representing > 1% of the salivary (Sa) microbiome, c) phylogenetic tree constructed from ASVs representing > 1% of the fecal (Fe) microbiome. Distinct phyla are represented by branches with different colors. Out of circle tree, red bars represent ASVs obtained from original samples' DNA (o-DNA), blue bars represent ASVs obtained from culturable samples' DNA (c-DNA), and green bars represent ASVs found in both original and culturable DNA samples' DNA (o-DNA and c-DNA); The outermost bars represent the proportion of each ASV in each microbiome.

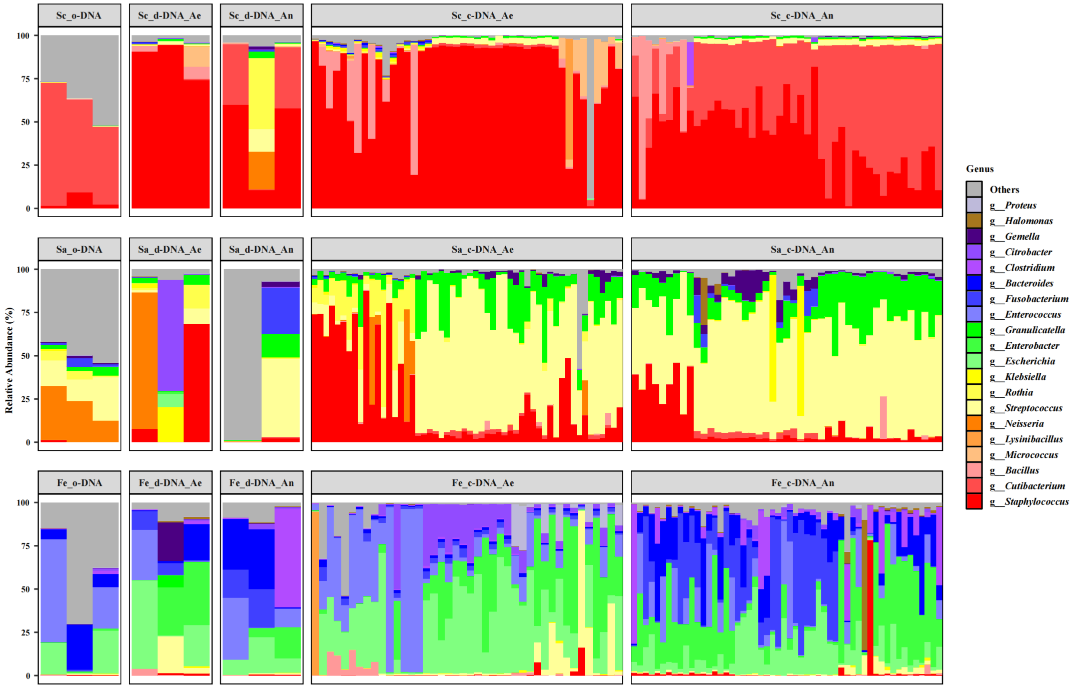

Supplementary Figure S3. Histograms of the top 20 genera found in the original and cultured samples' DNA. Direct-culture samples' DNA is indicated by d-DNA, and cultivation after simulated two-day transportation of samples' DNA is indicated by c-DNA. This specifically refers to culturable samples' DNA that do not include direct-culture samples' DNA. The other genera are represented as Others. Sc indicates scalp; Sa, saliva; and Fe, feces; and Ae and An indicate aerobic and anaerobic conditions, respectively.

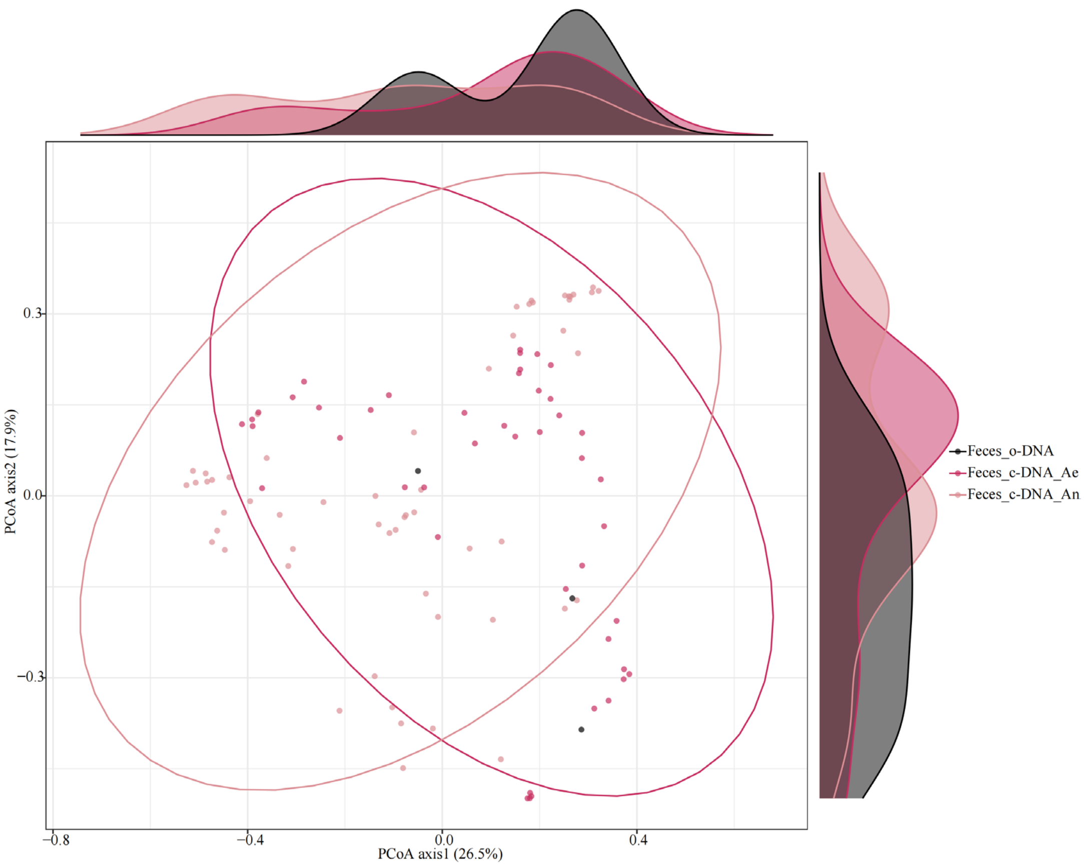

Supplementary Figure S4. PCoA plots based on Bray-Curtis dissimilarity showing the differences between original and culturable microorganisms at one site (feces) in the human body under aerobic and anaerobic conditions. Culturable samples' DNA is indicated by c-DNA. Aerobic and anaerobic conditions are indicated by Ae/An. The primer chosen for the fecal sample was V4 of the 16S rRNA gene; therefore, the results are not shown together with those of the scalp and fecal samples. Circles represent 95% confidence intervals for different c-DNA samples.

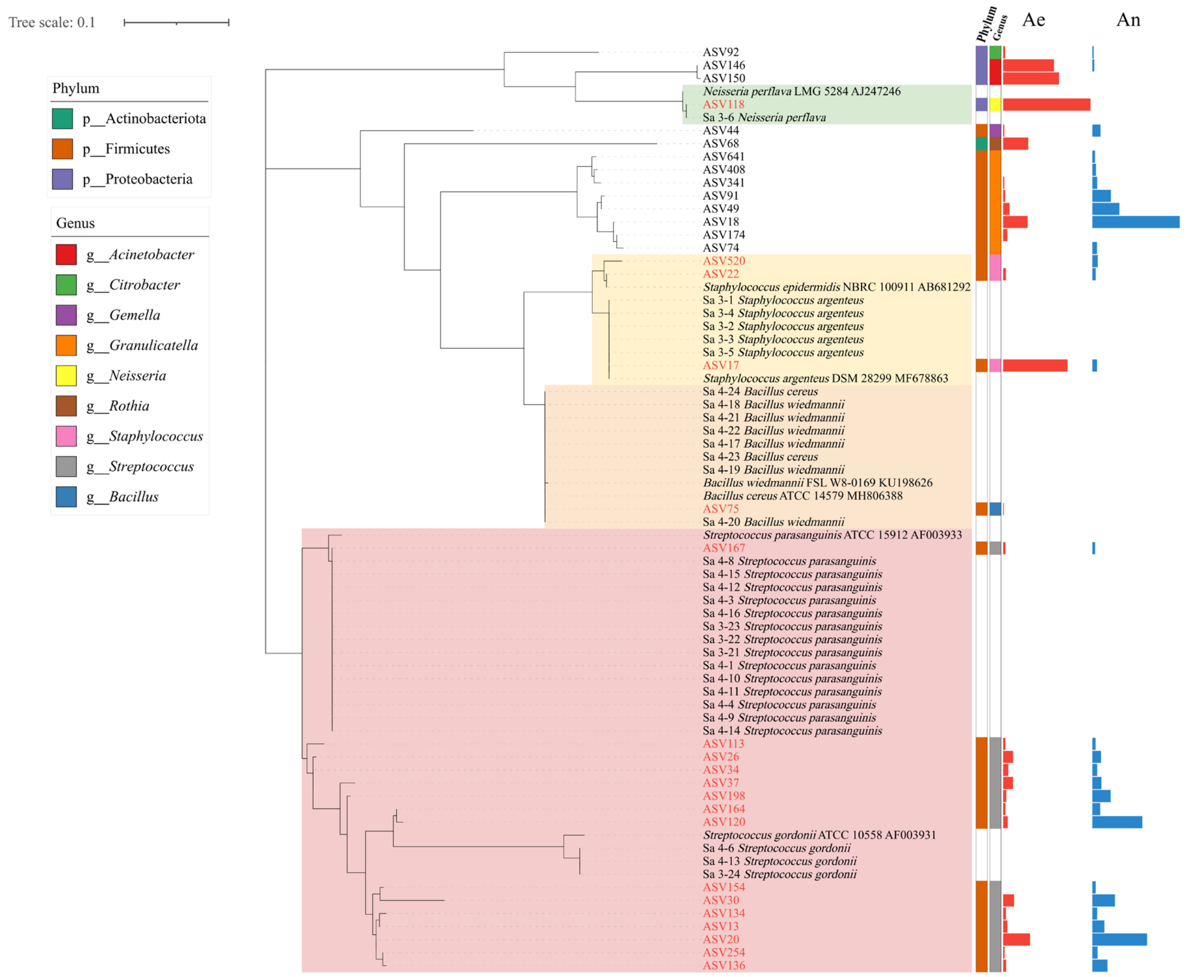

Supplementary Figure S5. Phylogenetic tree of the 16S rRNA gene constructed from the sequencing results of single strains isolated from saliva samples and major ASVs obtained through high-throughput sequencing. Phyla and genera are listed on the left. The columns labeled Ae/An represent the distribution of the major ASVs selected from the aerobic and anaerobic cultures. Labels beginning with Sa indicate the closest annotated results for the selected single salivary colony.

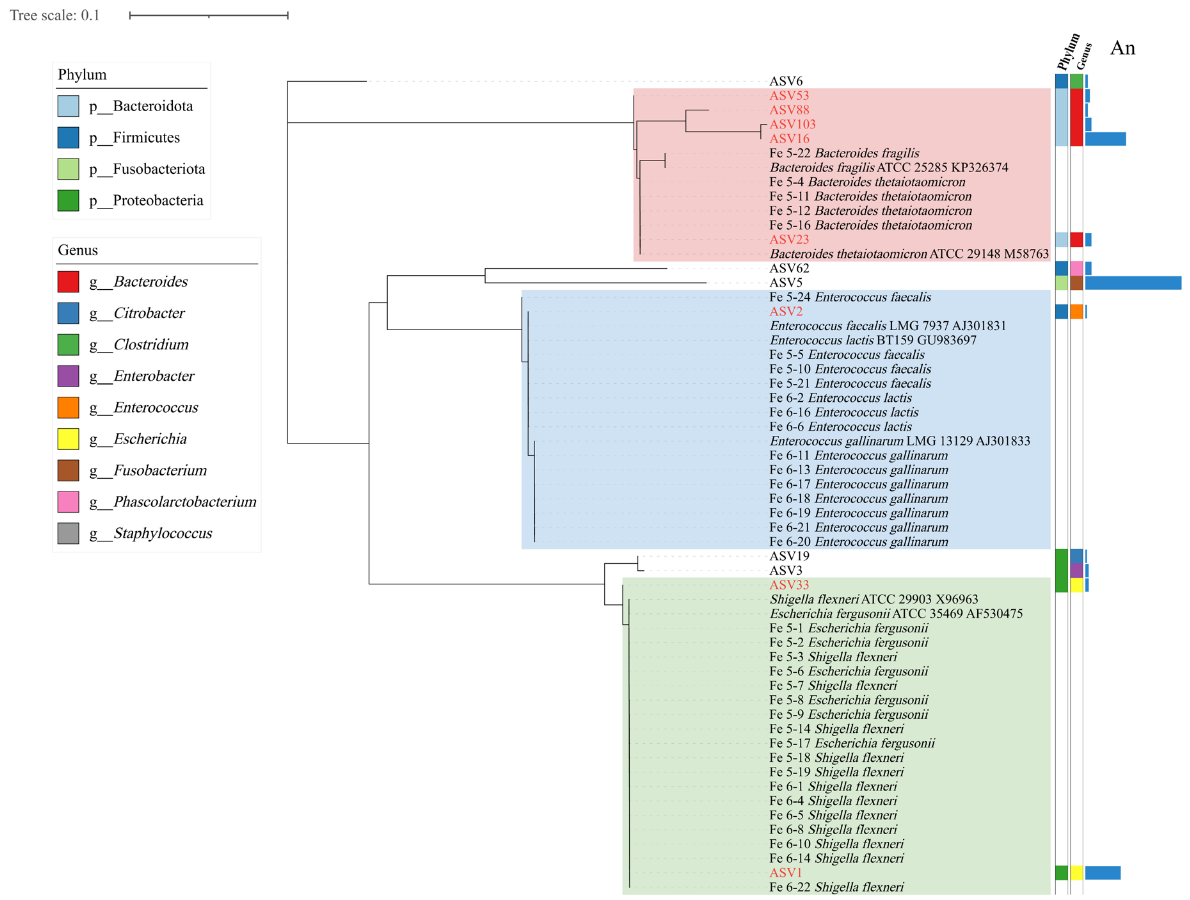

Supplementary Figure S6. Phylogenetic tree of the 16S rRNA gene constructed from the sequencing results of single strains isolated from feces cultivation samples and major ASVs obtained through high-throughput sequencing. Phyla and genera are listed on the left. The columns labeled An represent the distribution of the major ASVs selected from the anaerobic cultures. Labels beginning with Fe indicate the closest annotated result for the selected fecal colony.

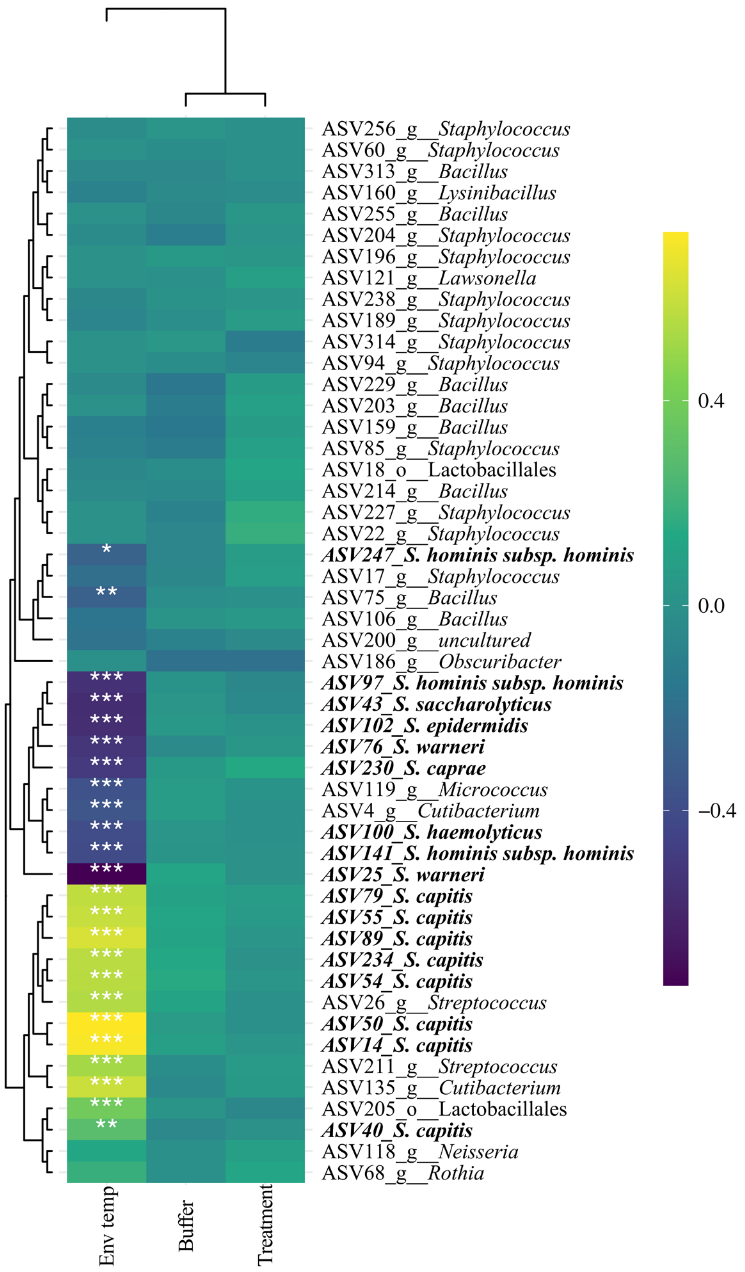

Supplementary Figure S7. Heatmap of the correlation analysis between transport temperature and culturable microorganisms from human scalp based on ASV-level analysis. The intensity of the color correlates with the temperature; blue hues indicate lower temperatures, whereas yellow hues indicate higher temperatures. ASVs related to *Staphylococcus* are highlighted in bold and were annotated at the species level based on single-colony results.

Appendix Tables

Supplementary Table S1: Medium used in the article.

Supplementary Table S2: Top 15 genera of original and culturable microorganisms in Scalp.

Supplementary Table S3: Top 15 genera of original and culturable microorganisms in Saliva.

Supplementary Table S4: Top 15 genera of original and culturable microorganisms in Feces.

Supplementary Table S5: Distribution of ASVs associated with *Staphylococcus* among the original and culturable microorganisms.

Supplementary Table S6: Distribution of ASVs associated with *Streptococcus* among the original and culturable microorganisms.

Supplementary Table S7: Colony counts of cultivable microorganisms from one scalp sample.

Supplementary Table S8: Colony counts of cultivable microorganisms from one saliva sample.

Supplementary Table S9: Colony counts of cultivable microorganisms from one feces sample.
